# PointTransformer: Encoding Human Local Features for Small Target Detection

**DOI:** 10.1155/2022/9640673

**Published:** 2022-08-21

**Authors:** Yudi Tang, Bing Wang, Wangli He, Feng Qian, Zhen Liu

**Affiliations:** ^1^Key Laboratory of Smart Manufacturing in Energy Chemical Process, Ministry of Education, East China University of Science and Technology, No. 130 Meilong Road, Shanghai 200237, China; ^2^Sinopec Shanghai Petrochemical Co., Ltd., No. 48 Jinyi Road, Shanghai 201512, China

## Abstract

The improvement of small target detection and obscuration handling is the key problem to be solved in the object detection task. In the field operation of chemical plant, due to the occlusion of construction workers and the long distance of surveillance shooting, it often leads to the phenomenon of missed detection. Most of the existing work uses multiple feature fusion strategies to extract different levels of features and then aggregate them into global features, which does not utilize local features and makes it difficult to improve the performance of small target detection. To address this issue, this paper introduces Point Transformer, a transformer encoder, as the core backbone of the object detection framework that first uses a priori information of human skeletal points to obtain local features and then uses both self-attention and cross-attention mechanisms to reconstruct the local features corresponding to each key point. In addition, since the target to be detected is highly correlated with the position of human skeletal points, to further boost Point Transformer's performance, a learnable positional encoding method is proposed by us to highlight the position characteristics of each skeletal point. The proposed model is evaluated on the dataset of field operation in a chemical plant. The results are significantly better than the classical algorithms. It also outperforms state-of-the-art by 12 percent of map points in the small target detection task.

## 1. Introduction

In recent years, the application of computer vision in chemical safety has developed rapidly. In the field operation of chemical plant, the most important element is safety, which often leads to very serious consequences due to the illegal construction by workers. With the development of deep learning, using this method to solve the safety problems in the field operation of chemical plant has become popular research nowadays. In surveillance video analysis, object detection algorithms, such as YOLO [1] and SSD [2], are often used to detect and identify construction sites using a large amount of training data, which can significantly improve the on-site safety protection level, as well as providing timely warnings for detected violations. However, in the field operation of chemical plant, the application scenario is very different from the traditional object detection task, where the equipment worn by workers needs more attention from the model because a large number of targets to be detected are highly relevant. It is difficult to solve this problem using classical object detection algorithms such as YOLO.

Many recent studies have introduced feature fusion modules [3] to improve the recognition rate of object detection algorithms in small targets and occlusion phenomena. By merging shallow local features and deep global features [4], the model can focus on both local features and global semantic information. These strategies have been widely used in object detection algorithms and have the potential to significantly improve the performance of algorithm on dataset, such as COCO [5]. In order to further improve the detection performance, many studies have introduced attention mechanism modules [6, 7] to reconstruct local features at occluded locations. By designing the attention mechanism, the model can make better use of local features in reasoning and determining the occluded regions. However, these strategies and improvements are only for the general scene application. They do not consider the special characteristics in the field operation of chemical plant. The objects to be detected are mainly focused on the construction workers, and how to extract the local features of the construction workers is the key to improving the recognition performance of our algorithm.

**Figure 1:**
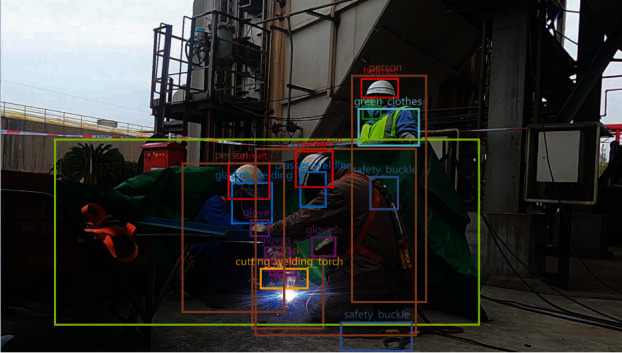
An example of labeling data, it can be seen from the figure that a large number of targets to be detected are highly correlated with construction personnel.

As shown in Figure 1, most of the targets to be detected in our research are highly related to construction workers and show a clear dependence on the skeletal point locations of workers, e.g., helmets are always worn on the head and gloves are always worn on the hand, which can be used as a priori knowledge for the detection task. Based on this, we use the trained OpenPose [8] model to extract 25 skeletal point positions of the human body as a priori information for the local features of subsequent model reconstruction. This local feature extraction method has been used to reconstruct human local features in many ReID [9, 10] studies. For example, Wang et al. [11] used human skeletal point features to solve the partial occlusion phenomenon, and inspired by this, human skeletal point local feature extraction will also be applied to our network structure.

First of all, in the backbone design, feature extraction methods such as traditional ResNet [12] and EfficientNet [13] are not used. Although these backbones have achieved excellent results in many classical challenges, the relatively deep network also impacts the construction of local features, which makes it difficult to improve the detection performance of small targets. We chose the popular transformer [14] architecture as the core feature extraction module to address this problem. Although the attention mechanism module can be used to reconstruct each local feature area better, most of the areas in our task are background, and we want the workers themselves to be given more attention by the model. Consequently, when the transformer module was designed, the method of gated positional encoding was introduced to focus on extracting local features in the human skeletal point region. Compared to the classic transformer architecture, we designed the module to focus on reconstructing features in the human skeletal points while downplaying irrelevant features such as the background.

Although the traditional self-attention [15] approach can reconstruct each part of the features by weight calculation when the attention mechanism module is designed, it is difficult to capture the interrelationship between the local features. In LoFTR [16], self-attention is used to reconstruct local features, and cross-attention is used to highlight the relationship between different key points. Inspired by this, the cross-attention method is also introduced to highlight the relational properties of different skeletal point regions when the human skeletal point region features are reconstructed. When construction workers work together, the tools and equipment they use are nearly the same, and the cross-attention approach also allows the characteristics of the construction scene and the collaborative work to be learned by the model.

Since transformer architectures are inherently insensitive to position information, it is often necessary to introduce positional encoding [17] features. Transformer architectures often use a fixed positional encoding method to highlight the characteristics of different regions, but these methods only give a unique identifier to each local feature region and do not have learning capabilities. In our study, most of the targets to be detected show obvious positional relationships. For example, the helmet must be at the top of the protective goggles. Based on this, a learnable positional encoding method is proposed by us. On one hand, the importance of position information is highlighted so that the model can better learn the position relationship between different objects. On the other hand, due to the different importance of human skeletal point features at different positions, for example, a large number of targets to be detected are concentrated on the hands and head, and a few on the human torso. Therefore, it is also possible to differentiate depending on the position of the target to be detected.

Based on our knowledge, there is currently no research on applying human skeletal points as local features in object detection algorithms for the field operation chemical plant scene. To solve the problem of small target detection and covering in this scene, we propose a novel end-to-end object detection framework with a transformer as the core backbone for feature extraction, and an improved attention mechanism is designed to highlight the relationship between local features. Additionally, since location information is particularly important in our research scenario, a learnable positional encoding method is also introduced to highlight the location relationship properties between the targets to be detected. The main contributions of our research are summarized as follows:

- A new type of end-to-end object detection backbone is proposed that optimizes the local feature extraction through the features of human skeleton points while designing and improving the attention module to improve the model's detection performance.
- Multiple attentional mechanisms are proposed to reconstruct the local features of human skeletal points and their interdependence information by using self-attention and cross-attention, respectively.
- In the transformer structure, a learnable positional encoding method is proposed to optimize the feature reconstruction of each local skeletal point by utilizing a weighting mechanism of the local features.

## 2. Related Works

### 2.1. Object Detection Models

This section introduces some fundamental concepts in the field of object detection and then elaborates and illustrates several popular attention mechanism modules and positional encoding methods.

### 2.2. Back Bone Design

The object detection algorithm is composed of four primary modules that were developed during the process. (1) Operations for data augmentation and preprocessing. (2) Design of backbone in feature extraction module. (3) Feature fusion module. (4) Output module. The data augmentation module is primarily used to increase the amount of training data and enhance the model's generalizability. The backbone is generally trained by classical classification models, such as those obtained by using ResNet on the ImageNet dataset, and the feature fusion module is mainly used to increase the diversity of features, such as the SPP [18] layer in YOLOV5. The output layer mainly uses the learned features to get the prediction results.

Almost all object detection algorithms perform data augmentation [19, 20]operations on the training data to expand the amount of data. For example, by applying CutMix operations to the data, the data is rotated and scaled in EfficientDet [21], and the overall Map is significantly improved. In YOLOV5 [19] Mosaic Data Augmentation operations are also used to increase the amount of training data. Utilizing data augmentation can significantly improve the model's generalizability and minimize the risk of overfitting.

Numerous object detection algorithms choose DenseNet [22, 23] and EfficientNet [13] as core feature extraction modules for backbone design. On one hand, these models perform well across all classical datasets. On the other hand, due to the abundance of pre-trained models, different pre-trained models can be selected based on the difference of application scenarios. However, because these models require more convolutional layers to achieve a larger receptive field, they tend to focus on global features and ignore some local features. As a result, the traditional backbone design method is better suited to large target detection tasks and will be significantly less effective at detecting small targets. Given that the majority of the targets in our task are related to construction workers and fall under the category of small target detection tasks, we will design and implement a new end-to-end network structure for the backbone selection.

### 2.3. Transformer Encoder

Both feature concatenation and fusion methods are widely used in the design of feature fusion modules, for example, the FPN [21, 24]method is used in mask R-CNN [25] to extract multiple layers of features, and SPP [18] is used in YOLOV5 to obtain richer features. The advantage of these methods is that they allow for the simultaneous use of deep and shallow features, which improves the model's detection performance of targets of various sizes. However, because these methods do not take into account the application scenario and do not select the appropriate features based on the characteristics of the detected target, we chose the transformer architecture, which is better suited for local feature reconstruction. The transformer architecture has demonstrated excellent performance in a variety of computer vision tasks, including object detection in DETR [26] using the transformer's encoder and decoder, and as the backbone of the Swin transformer [27, 28]in detection and classification scenarios, significantly improving model performance. The attention mechanism module is at the heart of the transformer architecture, as it recombines the features of each region by calculating the weight relationship between each local feature. The advantage of this approach is that the model can pay more attention to local features and also learn more about the relational properties between regions.

### 2.4. Positional Encoding Method

Due to the insensitivity of the transformer architecture to position information, additional position feature is typically added to highlight local features. BERT [29] uses a fixed positional encoding method to emphasize contextual information, while VIT [15] uses absolute positional encoding method to improve the model's classification performance. Typically, the obtained positional features must be fused with local features, which introduces position information into each local feature. However, because positional features are fixed, they cannot be updated for learning purposes, limiting their usefulness. In this task, we will enhance the positional encoding method in order to highlight the positional characteristics of various local features.

## 3. Methodology

**Figure 2:**
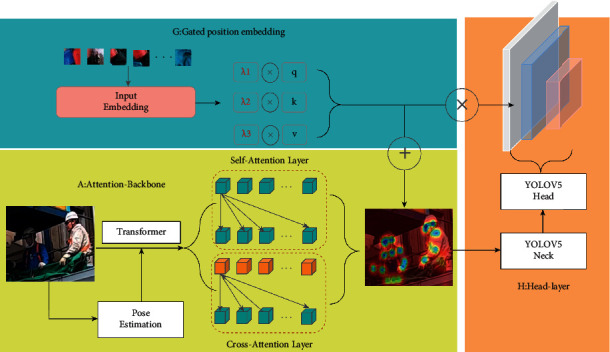
The overall architecture of our proposed model. Attention backbone (A) utilize a trained pose estimation model to reconstruct local features based on transformer encoder. Gated position embedding module (G) uses human skeletal point location information to enhance local feature learning. Head-layer module (H) reconstructs the output layer by weighting the positional encoding feature maps.

Our proposed framework is illustrated in Figure 2 and consists of the following points: a transformer encoder-based feature extraction backbone (A), which is used to extract features from the input image, mainly involving two attention mechanism modules, self-attention and cross-attention; a gated position encoding computation module (G), which is used to highlight the positional characteristics between different skeletal points; and a Head-Attention module (H), which uses the positional characteristics of skeletal points to enhance the detection effect of the output layer.

### 3.1. Revisiting Transformers and Small Target Detection Task

Classical object detection algorithms use ResNet, EfficientNet, etc. as the backbone [30] of feature extraction, which uses numerous layers of convolution in order to obtain a larger receptive field and then extracts features at different levels for the obtained different levels of feature maps. Although global features can be extracted at different scales by using operations such as FPN [24], the backbone with convolutional layers as the core is still insensitive to local features and it is difficult to obtain the relational properties between different regions.

The advantage of designing a backbone based on self-attention is that it can extract features for each local location; in turn, the problem of insensitivity to small targets in convolution is improved. The traditional transformer architecture first divides the input image according to a given region, for example, 16*∗*16 as the base unit for local feature reconstruction in VIT [15]. In order to use the transformer module to process the input image (**x** ∈ *ℝ*^*H*×*W*×*C*^), we reshape it and get the sequence input (**x**_*l*_ ∈ *ℝ*^*N*×(*L*^2^ · *C*)^), N represents the length of the sequence, and *L* represents the size of each token. But the problem of doing this is that if the input image is large and the selected window is small, it makes the computation inefficient, but if a larger window is set, it is difficult to mention the fine-grained extraction of local features, which has become one of the main problems of transformer framework nowadays. It is difficult to handle more fine-grained local feature extraction due to the limitation of computational magnitude. In our task, some large targets such as fire extinguishers and scaffolds are easily detected, which makes our main research problem focus on the construction workers' bodies, and these objects to be detected are usually highly correlated with human skeletal point locations. Based on this, the human skeletal point information will be used to highlight the characteristics of local features, thus allowing the model to focus more on small targets in the human body.

In the field operation chemical plant scene, if the application scenario involves only a single construction job, there is usually not much occlusion, which also makes the detection task relatively easy. However, in our task, it is almost always a multi-person collaborative work scenario of multi-people collaborative construction, which makes many local features easily obscured from each other. To solve the problem, inspired by LoFTR [16], both self-attention and cross-attention feature extraction methods are chosen to be applied to local feature computation, which can reconstruct local features by self-attention on one hand and extract relational properties between skeletal points by cross-attention on the other hand. For self-attention layers, the input features are key points at different locations of the same person. For cross-attention layers, the input features are key points that differ from person to person. All attention mechanism calculation methods are calculated by

(1) $$\begin{array}{c} \text{Attention}\left(Q,K,V\right)=\text{softmax}\left(QK^{T}\right)V, \end{array}$$

where *Q* indexes query vectors, *K* indexes key vectors, and *V* is the value vectors.

### 3.2. Local Feature Extraction

We first obtain the skeletal point positions of all construction workers by a trained pose estimation model [31, 32], 25 keypoints are obtained, all consisting of 2D coordinates. The feature map obtained after the backbone is expanded into a sequence for subsequent calculation of the attention mechanism module. The traditional transformer calculates self-attention on the entire sequence. However, in our task, we need to pay more attention to local features, that is, the regions corresponding to the key points. Based on this, we map the key points to the expanded sequence (downsampling ratio consistent with the backbone), which corresponds to part of the token in the corresponding sequence. In the calculation of self-attention, except for the tokens where the key points are located, the weights calculated from other positions are truncated, and the maximum is not over 0.05. The reason for this is that we do not want the model to consider too many background features. In the calculation of cross-attention, we design a mask mechanism, only the tokens corresponding to the key points will be updated. After the attention mechanism, we reshape the entire sequence to get its feature map (consistent with the size of the feature map in the last layer of the backbone).

A set of learnable weight parameters is designed by us to weight each local feature corresponding to each skeletal point. The reason for this is that most of the small targets to be detected in our dataset are concentrated on the hands and head, while the large targets to be detected are mainly on the torso. In order to improve the detection of small targets, we want the model to focus more on the hand and head locations and slightly on the body and leg locations. Based on this, we designed an additional set of learnable gated parameters to combine the local features and weighted the features at the location of skeletal points before calculating attention with other local features.

In the process of calculating self-attention, the difference with the original VIT method is that we weight the features at the locations of skeletal points and the weight parameters are learnable, which has the advantage of making the model more focused on the areas where small target objects exist, which is the core of our research problem. We do not use the same or random weights for the initialization of all skeletal points, but rather give larger weights for the hands and head, initialized to 10, and smaller weights for the body and leg key points, initialized to 2. For the location of other non-human skeletal points, it is consistent with the traditional transformer architecture. The self-attention method based on gated parameters allows the model to utilize more prior knowledge and focus on local feature extraction of the human body.

**Figure 3:**
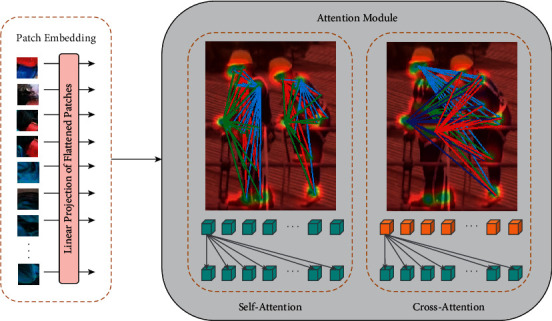
For each local feature, both self-attention and cross-attention mechanisms are used to reconstruct the local features of the human body.

When constructing local features, it is difficult to highlight the location relationship between skeletal points if only the self-attention method is used; for example, the helmet is always located above the glove location, and if one worker in the current construction scene is wearing gloves and helmet, all other workers should also be required to wear gloves and helmet. In chemical scenes, usually all workers in an area wear the same work equipment, but due to the small target and the existence of obscuration and other problems, there are frequently some missed tests phenomenon. In order to make full use of the positional features between objects, we additionally add the cross-attention module to optimize local feature extraction. As shown in Figure 3, for each skeletal point region of the construction worker, the attention between it and other construction workers' skeletal points is calculated in the same way as the traditional self-attention, and the superimposed features are averaged if there are multiple people in the figure. In the experiment, we will discuss the effects brought by these two attention mechanism modules separately.

### 3.3. The Prominent Role of Positional Encoding

**Figure 4:**
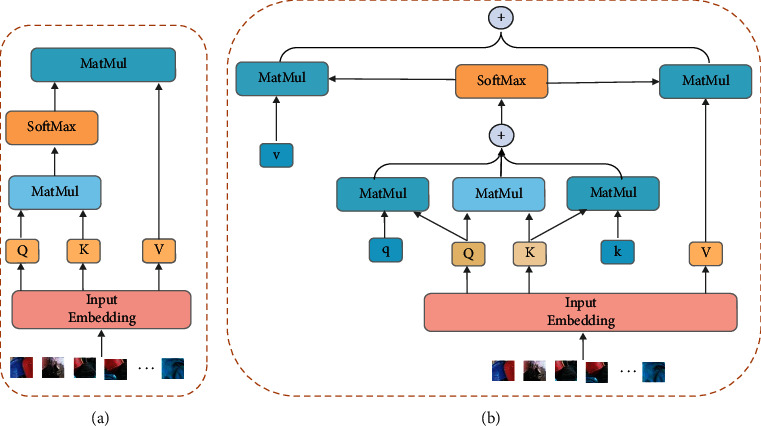
(a) The left figure shows the self-attention structure of the traditional transformer. (b) The right figure shows our proposed gated position-attention, which incorporates the influence of position information on the reconstructed features.

The advantage of using a transformer as a backbone for feature extraction is that it has strong reconstruction ability for local features, but such methods as self-attention are insensitive to positional information which only gives a unique identifier to each region and does not have an actual feature representation. In the field operation chemical plant scene, positional information is particularly important, e.g., tools are always held in the hands and safety buckles are always tied on the body, and there are obvious location characteristics between these objects and human skeletal points. Based on this, we introduced an additional learnable positional encoding method to highlight the importance of local features when designing the transformer architecture. This module is only for self-attention calculation and initializes the position encoding of the sequence expanded by the output feature map of the backbone. Different from the initialization method in VIT, the position encoding we designed is learnable, not a fixed parameter. In addition, it is not only related to its absolute position, but also needs to consider the characteristics of *K* and *V* corresponding to its token. Inspired by Wang et al. [17], for the positional encoding features as shown in Figure 4, we learn positional information for *Q*, *K*, and *V*, respectively, and its learned positional features are weighted together with the reconstructed features computed in self-attention, and the positional encoding method is computed in the same way as in Axial-DeepLab [17].

(2) $$\begin{array}{c} y=\sum_{p\in N_{m\times m}\left(o\right)}{\text{softmax}}_{p}\left(q_{o}^{T}k_{p}+q_{o}^{T}r_{p-o}^{q}+k_{p}^{T}r_{p-o}^{k}\right)\left(v_{p}+r_{p-o}^{v}\right), \end{array}$$

where *r*_*p*−*o*_^*k*^ ∈ *ℝ*^*d*_*q*_^ represents the learnable positional encoding for K, and *r*_*p*−*o*_^*v*^ ∈ *ℝ*^*d*_*out*_^ is the same for *V*.

In Medical Transformer [33], the positional encoding method is initialized randomly because the features at each location do not have a priori knowledge, but in our task, it is obvious that the location of human skeletal points has a more important role. Based on this, we do not choose a random approach when initializing the positional features, but perform a Gaussian initialization centered on each key point, which will result in a larger weight value for the region where the skeletal points are located and a smaller weight value for the other locations, which also matches the distribution of the objects to be detected in our task. Since the targets to be detected are highly concentrated in the hands and heads, we also give larger weight values when initializing their positional feature and the rest of the skeletal point locations are initialized with the same Gaussian initialization method.

### 3.4. Improvement of the Output Layer

**Figure 5:**
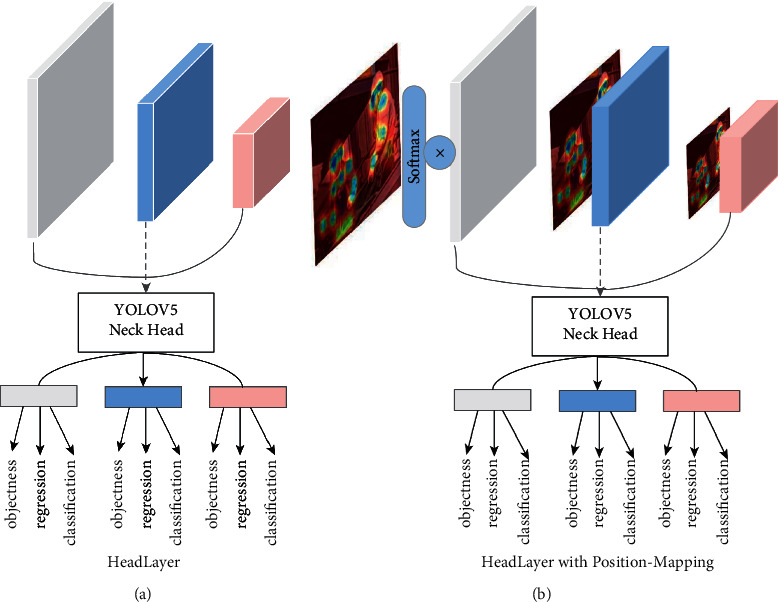
(a) The original YOLOV5 head-layer is shown on the left. (b) Our proposed head-layer with positional features mapping is shown on the right. The dependence of the model on local features is further enhanced by mapping the positional features to the output layer.

Since the position encoding feature is very sensitive to the features corresponding to the keypoints, we use a fully connected layer to its probability map to weight the output layer. In the object detection task, multiple anchor sizes and multiple output layers are usually designed to make the model adaptable to different size targets. Though the network structure is designed to focus on the attention method and emphasize the importance of positional information, the local features corresponding to the skeletal points cannot be well utilized if the output layer is still chosen similar to the YOLOV5 head-layer, which only predicts the features at different levels separately, so we perform an additional weighting calculation for the output features. As presented in 3.3, the learnable positional encoding features are multiplied with each output layer feature in YOLOV5 as shown in Figure 5. This enables more attention to be paid to the human skeletal point area; thus, improving the detection performance.

## 4. Experiments

In this chapter, we evaluate our proposed method in the field operation chemical plant scene. We set up several sets of ablation experiments and analyze them in comparison with the corresponding performance of the mainstream object detection algorithms presently. We will present the experimental setup and results in the following sections.

### 4.1. Datasets Description

The data we selected came from the scene of field operation chemical plant, and because the surveillance video was blurred, so we chose to shoot the construction site in person. All videos were shot with 1080p explosion-proof equipment. To make the data more diverse, we chose different angles and distances for the same construction scene. All video data were cut into images at 100 frames intervals to build a dataset and annotated it, and all data were manually annotated using the LabelMe toolkit. The overall dataset consists of 2400 annotated images, but since our research focuses on the presence of small targets and occlusions, some easy-to-detect data samples were excluded from the original dataset. The dataset was randomly split where the training set consisted of 1281 images and the test set consisted of 200 images. For the selection of labels, there are 19 categories of labeled objects in total, including helmets, goggles, gloves, construction equipment, fire extinguishers, and signs. However, since objects such as fire extinguishers and scaffolds are usually easier to be detected, we only kept the objects related to construction workers in the labels, and the total number of labels is 13.

### 4.2. Influence of Different Attention Methods

In this part, we conduct a comparative experimental analysis of different design approaches for the attention mechanism module. In the next experiments, we choose the evaluation method consistent with the COCO benchmark [5]. Firstly, all human skeletal point locations are obtained using the OpenPose model. Next in the backbone selection, we conducted the following experiments, respectively: (1) directly using the native YOLOV5 model; (2) using only the basic transformer encoder as the feature extraction backbone; (3) using both self-attention and cross-attention as the feature extraction backbone. In the above experiments, all attention mechanism modules do not use positional encoding features.  Table 1 shows that if only the traditional object detection algorithm is used, it is difficult to get better performance in the dataset with mostly small targets, and when using transformer as the backbone, although the detection effect can be slightly improved on small target objects, the overall map value is not significantly improved. When both self-attention and cross-attention modules are used, the detection performance is not only improved by 5.6 percentage points on small targets, but also the overall map value is improved by 3.1 percentage points. This can be attributed to the fact that by using multiple attention mechanism strategies allows the model to learn richer and more reliable local features, and by cross-attention also allows the model to learn the relational properties between different local features.

### 4.3. Influence of Position Encoding Method

Since the targets to be detected shows an obvious dependence on the location of human skeletal points, we designed a learnable positional encoding method. In the next experiments, we will analyze the performance of different positional encoding methods on the results which are used with self-attention and cross-attention as the base backbone. First, we used the traditional transformer positional encoding method that is consistent with VIT, and Table 2 shows that the introduction of positional encoding method can increase the overall Ap value of the model by up to 1.3 percentage points, which has a significant impact on the detection performance. Furthermore, the traditional positional encoding method was replaced with a learnable gated positional encoding method, and the gated values (weights) were initialized to 0.05 for *Q*, *K*, and *V*. Since the method of positional encoding is randomly initialized at the beginning of training, which may lead to instability occurred during model training, on the basis of that, a smaller initial value was chosen for this parameter. From Table 2, it can be concluded that the use of our proposed gated positional encoding method can improve the overall detection performance by 2.4 percentage points. On the effect of detection for small target objects, the improvement is 1.9 percentage points relative to the traditional positional encoding method. This can be attributed to the fact that, for the detection task of the equipment worn by the construction personnel, a large number of targets to be detected show obvious characteristics of positional relationships, and by training the learnable positional encoding method, the model can better learn the positional dependencies between different objects.

### 4.4. Influence of the Output Layer

In this part, we will compare and analyze the effect of the output layer of the object detection algorithm on the results. Three output layers are selected in YOLOV5 for regression and classification tasks after concat features from different layers, respectively, using different receptive field features for the prediction of different size targets. In our design, positional-encoded weight mapping is additionally introduced on top of it to further highlight the degree of influence of different skeletal point locations on the results in the output layer. From Table 3, it can be seen that the output layer with the positional-encoded weight mapping improves the detection of small targets by 1.7 percentage points. This can be attributed to the fact that, although multiple attention and location encoding strategies are used in the backbone module, some features and information are lost if not emphasized in the output stage. The combination of positional-encoded weight mapping with the output layer can significantly improve the detection performance of our model for small target detection tasks.

### 4.5. Comparison with the SOTA Model

To highlight the effectiveness of our proposed method, in this chapter, we will compare and analyze our method with the current SOTA object detection algorithms. In order to analyze the effectiveness of the transformer module in the field operation of chemical plant, we conduct an experimental comparison with EfficientDet and FCOS. Since the detection performance of EfficientDet is directly related to the levels of EfficientNet, we select EfficentNet-B0 and EfficentNet-B3 as backbones to observe their effect on small target detection task. In order to prove the importance of local features in the small target detection task, we choose to compare with the Transformer-based Deformable DETR method and select ResNet50 as its backbone. In the data preprocessing stage, all models use the same data augmentation strategy, and for the fairness of the experiment, all models do not use multi-scale input, and all input sizes are 640*∗*640. Due to the instability of the label balancing method during training, we did not use this method for all models. From Table 4, it can be seen that although the EfficientDet model has a good performance in large target detection, it cannot effectively identify small targets. In addition, when the levels of backbone layers increased, the small target detection performance is not improved. Although the transformer is used as the entire encoder and decoder modules in Deformable DETR, there are still problems in the small target detection task in the field operation of chemical plant. Even if two-stage training is performed on Deformable DETR, it is difficult to improve its small target detection performance. Through the above comparison experiments, it can be found that for the small target detection problem in the field operation of chemical plant, not only the feature relationship between regions needs to be considered in the selection of network structure, but also the local feature extraction module is required to strengthen the model's local perception ability.

### 4.6. Training Details

The overall training process of the model is consistent with YOLOV5, using ADAM [34] as the optimizer and choosing a moment value of 0.9, an initial learning rate of 0.01, and a learning rate decay and early stop strategy. All network structures are used in YOLOV5-X structure except backbone design. All experiments are based on the same evaluation criteria used in the COCO dataset after 300 epochs of iterations of RTX3090.

## 5. Conclusions

In the field operation of chemical plant, there are often small target detection tasks and construction workers obscure each other. How to perform local feature extraction becomes the key to improve the detection performance. To solve this problem, we propose the point transformer, which first uses self-attention and cross-attention for local feature reconstruction of human skeletal points. In addition, since the target to be detected in our task is highly correlated with the location of the skeletal points of the construction workers, we designed a learnable positional encoding method to highlight the importance of location information in order to make better use of this priori information. It is shown in experiments on the scene of field operation chemical plant dataset that the proposed point transformer outperforms present-day classical object detection algorithms. Our approach can be seen as an application of optimizing the performance of small target detection tasks using local features of the human body. However, this has not yet been exploited due to the obvious synergistic relationship between the movement changes of the skeletal points during the construction work, which exhibits graph structural properties. Our future work will aim at using the graph model to construct local features of the human body to further improve the detection performance [35].

## Figures and Tables

**Table 1:** Influence of different backbone design on feature encoding.

Backbone design	AP	AP50	AP75	APs	APm	APl
YOLOV5-X	31.4	40.1	33.2	17.3	38.7	53.5
Transformer encoder	31.6	40.4	33.7	18.6	39.6	49.8
Self-attention and cross-attention	34.5	42.1	37.1	24.2	42.1	45.6

**Table 2:** Ablation studies of positional encoding method.

Positional-encoding method	AP	AP50	AP75	APs	APm	APl
Without positional encoding	34.5	42.1	37.1	24.2	42.1	45.6
2D positional encoding	35.8	43.6	38.9	25.7	42.2	45.1
Learnable gated positional encoding	36.9	45.1	40.3	27.6	44.1	44.4

**Table 3:** Influence of positional-feature-mapping on head-layer.

Head-layer type	AP	AP50	AP75	APs	APm	APl
YOLOV5-X head-layer	36.9	45.1	40.3	27.6	44.1	44.4
Positional-feature-mapping head-layer	37.2	45.5	40.7	29.3	42.1	43.2

**Table 4:** Comparison with the SOTA model.

Method	AP	AP50	AP75	APs	APm	APl
FCOS	28.1	39.5	31.3	16.5	35.7	48.1
EfficentNet-B0-based EfficientDet	27.9	38.6	30.8	16.8	34.5	46.6
EfficentNet-B3-based EfficientDet	30.8	40.8	33.6	17.0	36.2	49.1
Transformer-based Deformable DETR	33.5	42.7	36.1	19.4	41.5	51.7
YOLOV5-X	31.4	40.1	33.2	17.3	38.7	53.5
Our proposed model	37.2	45.5	40.7	29.3	42.1	43.2

## Data Availability

The data set was taken on site during the construction of Sinopec Shanghai Petrochemical. Due to its confidentiality policy, this dataset cannot be made public.
